# Supplementary material for: Nicotinamide riboside is uniquely and orally bioavailable in mice and humans
Source: Nat Commun. 2016 Oct 10;7:12948. doi: 10.1038/ncomms12948 (PMC5062546; doi:10.1038/ncomms12948)
Supplement: Supplementary Information — Supplementary Figure 1 and Supplementary Tables 1-4 [file ncomms12948-s1.pdf]

Supplementary Figure 1. Hepatic NR, NAR, and Me2PY concentrations after gavage of NR, Nam and NA

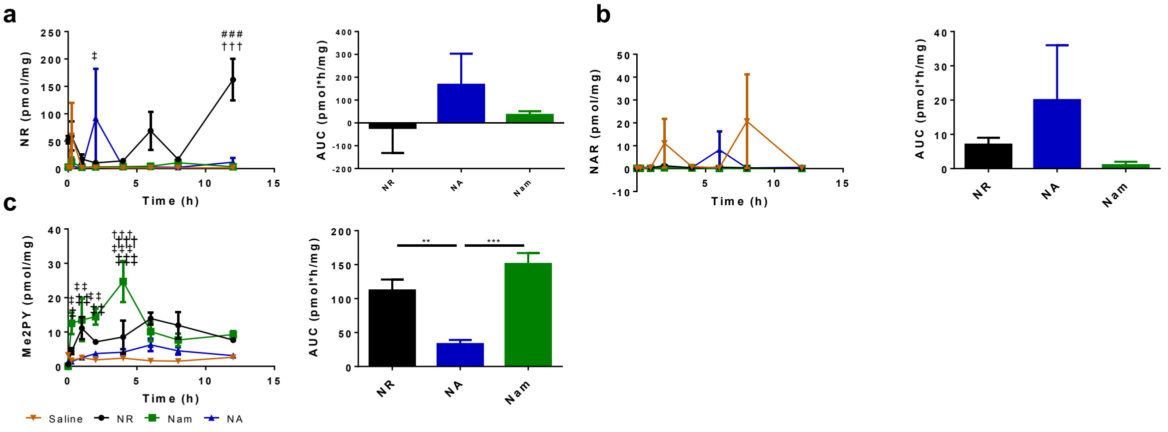

Either saline or equivalent moles of NR, NA or Nam were administered to male C57BL6/J mice by gavage. To control for circadian effects, gavage was performed at indicated times prior to a common ~2 pm sacrifice. In the left panels, the hepatic concentrations of each metabolite (pmol per mg of wet tissue weight) are shown as a function of the four gavages. In the right panels, the baseline subtracted 12-hour areas under the curve are shown. Left panels: ‡ p-value < 0.05; ‡‡ p-value < 0.01; ‡‡‡ p-value <0.001 Nam vs NA; † p-value < 0.05; †† p-value < 0.01; ††† p-value < 0.001 Nam vs NR; # p-value < 0.05; ## p-value < 0.01; ### p-value < 0.05 NA vs NR; Right panel: * p-value < 0.05; ** p-value < 0.01; *** p-value <0.001.

Supplementary Table 1. PBMC NAD^+ metabolites (μM) in a 52 year-old male who orally ingested 1000 mg NR Cl for 7 consecutive days

Time (hr)	NAD^+	$NADP^+$	NaAD	NMN	ADPR	Nam	MeNam	Me4PY	Me2PY
0	12	9.1	<0.02	0.68	0.70	3.2	0.06	0.20	1.1
0.3	27	23	<0.02	1.8	3.3	4.5	0.14	0.44	2.2
0.6	17	13	<0.02	1.4	2.1	7.1	0.08	0.57	3.1
1	24	13	<0.02	1.3	1.3	3.2	0.20	0.59	3.4
1.4	19	11	<0.02	1.1	1.3	3.7	0.15	0.64	3.1
2.7	12	6.9	<0.02	0.77	0.85	2.9	0.13	0.53	2.8
4.1	42	27	<0.02	2.7	3.4	4.9	0.32	1.9	11
7.7	43	27	0.51	2.6	2.5	5.9	0.49	3.5	17
8.1	50	33	0.91	2.9	3.5	5.7	0.60	4.4	22
23.8	32	17	0.37	1.9	2.5	2.6	0.25	1.9	8.8
167.6	38	12	0.45	1.1	2.1	3.2	0.66	2.5	11

Supplementary Table 2. Plasma NAD^+ metabolites (µM) in a 52 year-old male who orally ingested 1000 mg NR Cl for 7 consecutive days

Time (hr)	Nam	MeNam	Me4PY	Me2PY
0	0.70	0.10	0.60	2.2
0.6	0.90	0.40	1.1	5.7
1	1.2	0.60	1.4	5.7
1.4	1.1	0.50	1.4	6.6
2.7	0.90	0.60	1.8	7.5
4.1	1.2	0.80	2.6	10
7.7	1.1	0.70	3.2	13
8.1	0.60	0.90	3.8	14
23.8	0.80	0.90	3.9	14
167.6	1.5	2.1	4.6	17

Supplementary Table 3. Urinary NAD^+ metabolites (µmol/mmol creatinine) in a 52 year-old male who orally ingested 1000 mg NR Cl for 7 consecutive days

Time (hr)	Nam	MeNam	Me4PY	Me2PY
Pre-1st dose	0.26	5.5	2.8	13
0 - 4.1	0.16	6.5	3.8	20
4.1 - 7.7	2.9	26	21	180
8.1 - 12	1.8	26	24	99
Pre-2nd dose	1.4	29	29	108
Pre-5th dose	1.2	30	24	80

Supplementary Table 4. NAD^+ Metabolite AUC Increases as a Function of Single Doses of NR

Metabolite	NR Dose (mg)	AUC Increase from Baseline (%)			Comparison (Tukey Test)	
		Mean	SEM	N	vs. 100 mg	vs. 300 mg
NMN	100	13	14	8		
	300	47	38	10	ns	
	1000	23	15	8	ns	ns
NAD^+	100	48	15	12		
	300	59	40	12	ns	
	1000	92	49	12	ns	ns
Nam	100	14	7	12		
	300	19	21	12	ns	
	1000	16	10	12	ns	ns
MeNam	100	12	20	9		
	300	63	22	11	ns	
	1000	231	42	9	***	**
me2PY	100	53	14	10		
	300	201	88	9	ns	
	1000	609	146	9	**	*
NAAD	100	705	323	10		
	300	1287	480	10	ns	
	1000	2873	1250	10	*	ns

* P < 0.05, ** P < 0.01, *** P < 0.001
